# Machine learning applied to ambulatory blood pressure monitoring: a new tool to diagnose autonomic failure?

**DOI:** 10.1007/s00415-022-11020-2

**Published:** 2022-02-22

**Authors:** Fabrizio Vallelonga, G. Sobrero, A. Merola, M. Valente, M. Giudici, C. Di Stefano, V. Milazzo, J. Burrello, A. Burrello, F. Veglio, A. Romagnolo, S. Maule

**Affiliations:** 1 Autonomic Unit and Hypertension Unit, Internal Medicine Division, Department of Medical Sciences, University of Turin, via Genova 3, 10126 Turin, Italy; 2 Department of Neuroscience “Rita Levi Montalcini”, University of Turin, via Cherasco 15, 10124 Turin, Italy; 3 Department of Neurology, Wexner Medical Center, Ohio State University, Columbus, OH USA; 4 Department of Electrical, Electronic and Information Engineering “Guglielmo Marconi” (DEI), University of Bologna, Bologna, Italy

**Keywords:** Supervised learning, Linear discriminant analysis, Autonomic failure prediction

## Abstract

**Background:**

Autonomic failure (AF) complicates Parkinson’s disease (PD) in one-third of cases, resulting in complex blood pressure (BP) abnormalities. While autonomic testing represents the diagnostic gold standard for AF, accessibility to this examination remains limited to a few tertiary referral centers.

**Objective:**

The present study sought to investigate the accuracy of a machine learning algorithm applied to 24-h ambulatory BP monitoring (ABPM) as a tool to facilitate the diagnosis of AF in patients with PD.

**Methods:**

Consecutive PD patients naïve to vasoactive medications underwent 24 h-ABPM and autonomic testing. The diagnostic accuracy of a Linear Discriminant Analysis (LDA) model exploiting ABPM parameters was compared to autonomic testing (as per a modified version of the Composite Autonomic Symptom Score not including the sudomotor score) in the diagnosis of AF.

**Results:**

The study population consisted of *n* = 80 PD patients (33% female) with a mean age of 64 ± 10 years old and disease duration of 6.2 ± 4 years. The prevalence of AF at the autonomic testing was 36%. The LDA model showed 91.3% accuracy (98.0% specificity, 79.3% sensitivity) in predicting AF, significantly higher than any of the ABPM variables considered individually (hypotensive episodes = 82%; reverse dipping = 79%; awakening hypotension = 74%).

**Conclusion:**

LDA model based on 24-h ABPM parameters can effectively predict AF, allowing greater accessibility to an accurate and easy to administer test for AF. Potential applications range from systematic AF screening to monitoring and treating blood pressure dysregulation caused by PD and other neurodegenerative disorders.

## Introduction

Autonomic failure (AF) complicates Parkinson’s disease (PD) in up to one-third of cases. Cardiovascular AF disrupts neural networks controlling blood pressure (BP) and heart rate (HR), resulting in complex abnormalities in BP control, such as orthostatic hypotension (OH), supine hypertension (SH), abnormal circadian rhythm, and increased BP variability (BPV) [1]. These abnormalities are usually asymptomatic and difficult to recognize by clinical assessment alone [2, 3]. Still, they may result in organ damage [4] and functional disability [5], leading to greater morbidity and quality of life impairment [6], as well as worse clinical prognosis [7].

Unfortunately, accessibility to cardiovascular autonomic reflex testing (CART), the gold standard for diagnosing AF, is limited due to the complexity of the examination, technical skillset, and expensive equipment required to carry out this complex diagnostic test [8]. As a result, only patients complaining of “classic” OH symptoms, such as postural light-headedness or fainting, are usually referred to CART, and the execution of the test may require long travels to highly specialized tertiary referral centers.

Recent studies showed that selected abnormalities in the 24-h BP profiles, such as a reversed circadian rhythm [9] and increased BPV [10], are associated with AF. The central hypothesis of the present study is that ABPM effectively predicts adrenergic AF in patients with PD. To test this hypothesis, a prospective non-interventional study was designed to evaluate the diagnostic accuracy of a machine-learning algorithm of ABPM recordings compared to standard adrenergic autonomic testing in a cohort of consecutive PD patients.

## Methods

Consecutive patients referred to the Autonomic Unit of the Department of Medical Science, University of Torino (Italy) between September 2016 and June 2019 were offered to participate in a single-centre, cross-sectional study investigating the diagnostic potential of a machine-learning algorithm applied to ABPM as a tool to diagnose AF in PD.

### Inclusion criteria

Diagnosis of PD as per the EFNS/MDS-ES recommendations [11] for at least 2 years; stable dosage of dopaminergic drugs for at least 4 weeks.

### Exclusion criteria

Other neurological diseases associated with primary AF (multi-systemic atrophy, pure autonomic failure); diabetes mellitus or diseases potentially associated with secondary AF [12]; non-sinus rhythm or pacemaker-guided cardiac activity; severe cognitive impairment, defined as Montreal Cognitive Assessment (MoCA) score < 21 [13], or any physical impairment preventing the execution and interpretation of CART; a medical history of severe impaired renal function, heart diseases, or obstructive sleep apnoea syndrome; and ongoing vasoactive therapy (anti-hypotensive and/or anti-hypertensive) for orthostatic hypotension and/or supine hypertension.

### Study protocol

After the acquisition of written informed consent, those meeting all the inclusion and none of the exclusion criteria underwent CART followed by 24 h-ABPM within 10 days.

#### CART: technical execution

Autonomic testing has been performed as per a standard procedure and cardiovagal and adrenergic indexes calculated according to a modified version of the Composite Autonomic Symptom Score (CASS), without the sudomotor score [14]. Briefly, BP and the HR interval were continuously recorded using a beat-to-beat non-invasive monitor (Finometer, Finapres) during the performance of the following standardized tests:

1. Deep breathing: patients were asked to breathe deeply and evenly at 6 breaths/min for 1 min.
2. Valsalva manoeuvre: patients were asked to blow into a mouthpiece attached to an aneroid pressure gauge at a pressure of 40 mmHg, for 15 s.
3. Head-up tilt test: patients were asked to lye supine on the tilt table for 10 min, then the table was tilted up to a 60° upright position for 5 consecutive minutes. For this test, in addition to the beat-to-beat recording, the BP was measured with an automatic sphygmomanometer (Omron, HEM-9219 T-E, Japan©) at baseline, 1 min, 3 min, and 5 min

BP and HR variations were analysed with dedicated software (DAN Test Microlab, Padua, Italy) and scored using age-related normal ranges [15].

#### CART: data interpretation

OH was defined as a sustained reduction of systolic blood pressure ≥ 20 mmHg or diastolic blood pressure ≥ 10 mmHg within three minutes from standing [16].

SH was defined as systolic blood pressure ≥ 140 mmHg and/or diastolic blood pressure ≥ 90 mmHg recorded after at least 5 min of supine rest [3].

AF was diagnosed when the sum of the cardiovagal and adrenergic score was ≥ 2.

#### ABPM: technical execution

24-h ABPM were performed using a Spacelabs portable device (Spacelabs 90207—Spacelabs Inc., Redmond, WA, USA©) with appropriately sized arm-cuff placed on the non-dominant side, as per current guidelines [17]. BP was measured every 15 min during both daytime and nighttime; patients were asked to record on a diary relevant behavioural and occupational activities, sleep and wake time, and meals.

#### ABPM: data interpretation

ABPM was performed according to definitions and reference values for ABPM data interpretation proposed by the European Society of Hypertension [17]. Specifically, the following parameters were derived:

- BP load, defined as the percentage of blood pressure values exceeding reference values during daytime (≥ 135/85 mmHg) and nighttime (≥ 120/70 mmHg).
- Reverse dipping, defined as a systolic day-night difference ≤ 0 mmHg (i.e., average nocturnal systolic BP higher than average diurnal systolic BP).
- Weighted blood pressure variability (w-BPV), defined as the sum of the standard deviation of diurnal and nocturnal systolic BP, normalized for daytime and night-time duration. W-BPV was considered increased when > 11 [18].
- Postprandial hypotension (PPH), defined as a reduction in systolic blood pressure ≥ 20 mmHg within 120 min after a meal, using the mean of the last three BP measurements before the meal as reference [19].
- Hypotensive episodes, defined as any record of systolic BP values lower than average 24-h systolic BP by at least 15 mmHg between awakening and lunch time (Hypo-ep^Δ15/24h^) [20].
- Awakening hypotension, defined as the presence of at least one Hypo-ep ^Δ15/24h^ within 90 min from awakening (Hypo-aw^Δ15/24h^) [20].

### Statistical analysis

Analyses were performed with SPSS (Statistical Package for the Social Sciences—version 22—© 2014 IBM). Normal distribution of continuous variables was tested using the Shapiro–Wilk test. Continuous variables were expressed as mean ± standard deviation. Qualitative variables were expressed as absolute values of frequency and percentage values. Differences between two independent groups were evaluated using Student’s *t*-test for continuous variables with normal distribution and Mann–Whitney test for continuous variables with non-normal distribution; multiple comparisons (between more than 2 groups) were evaluated with One-way ANOVA analysis and Bonferroni’s correction. Categorical variables were compared using chi-square test or Fisher’s exact test according to the sampling number of analysed groups.

Univariate logistic regression analysis was used to evaluate the correlation between selected categorical ABPM abnormalities and AF; subsequently, multivariate logistic regression was performed to correct for age, sex, LEDD and disease duration. *P* values less than 0.05 were considered statistically significant.

#### Diagnostic accuracy of single ABPM parameters

For categorical variables, 2 × 2 contingency tables were built setting ABPM parameters as a diagnostic test and the presence of AF as the real outcome. Sensitivity, specificity, positive predictive value (PPV), and negative predictive value (NPV) were then calculated.

For continuous variables, a receiver operating characteristic (ROC) analysis was used to estimate the predictive accuracy (state variable: presence of autonomic failure; test variable: ABPM continuous parameters). Sensitivity, specificity, PPV, and NPV were calculated after the selection of the optimum ROC cut point, based on the balance between sensitivity and specificity (highest Youden index).

#### Global ABPM diagnostic accuracy: linear discriminant analysis

Supervised machine learning algorithms were trained using Python 3.5 (library, scikit-learn). Linear discriminant analysis (LDA) [21, 22] was applied to develop a prediction model for AF in PD based on ABPM data. LDA employs linear combinations of variables to maximize the separation between groups by increasing precision estimates by variance reduction. The algorithm computes a set of coefficients for a linear combination of each variable to predict the diagnosis of AF. The estimation is derived from the following equation: AF diagnosis = LDA_coeff1_ × variable_1_ + LDA_coeff2_ × variable_2_ + … + LDA_coeffn_ × variable_*n*_ > cut-off. The presence/absence of AF was set as an outcome; the following variables were used to train the model: 24-h, daytime and nighttime blood pressure values (systolic, mean, and diastolic), 24-h, daytime and nighttime blood pressure standard deviations (SD), daytime and nighttime blood pressure loads (systolic and diastolic), w-BPV, PPH, reverse dipping, Hypo-aw^Δ15/24h^, number of Hypo-ep^Δ15/24h^.

## Results

The study population consisted of 80 PD patients, 54 males (67.5%) and 26 females (32.5%), with a mean age of 64 ± 10 years, and PD duration of 6.2 ± 4 years. All patients were treated with dopaminergic drugs with a levodopa equivalent daily dose (LEDD) of 668 ± 351 mg [23].

According to the CART assessment, 29 patients (36%) were diagnosed with AF (AF+). This group was older but had similar disease duration and LEDD compared to the group without AF (AF−). Night-time average BP and BP loads were higher in patients AF+. Also, this group showed a higher incidence of reverse dipping, increased SD of systolic daytime BP, and hypotensive episodes compared to AF− (Table 1).

**Table 1:** ABPM parameters: comparison between patients with and without autonomic failure

	Ambulatory blood pressure monitoring			
	AF− (*n*. 51)	AF+ (n. 29)	*P* value	
Age (years) (mean ± SD)	61 ± 10	67 ± 10	< 0.01	
Disease duration (years) (mean ± SD)	5.5 ± 3	7 ± 4.5	0.08	
LEDD (mg) (mean ± SD)	657 ± 326	694 ± 403	0.72	
Female sex [*n* (%)]	17 (33)	9 (31)	0.08	
Daytime SBP (mmHg) (mean ± SD)	122 ± 10	118 ± 8	0.04	
Daytime MBP (mmHg) (mean ± SD)	91 ± 9	88 ± 7	0.24	
Daytime DBP (mmHg) (mean ± SD)	75 ± 9	73 ± 7	0.13	
Night-time SBP (mmHg) (mean ± SD)	109 ± 11	122 ± 17	< 0.01	
Night-time MBP (mmHg) (mean ± SD)	79 ± 8	89 ± 14	< 0.01	
Night-time DBP (mmHg) (mean ± SD)	64 ± 8	71 ± 13	< 0.01	
Daytime SBP loads (%) (mean ± SD)	19 ± 20	15 ± 11	0.19	
Daytime DBP loads (%) (mean ± SD)	17 ± 22	18 ± 15	0.83	
Night-time SBP loads (%) (mean ± SD)	19 ± 24	46 ± 36	< 0.01	
Night-time DBP loads (%) (mean ± SD)	23 ± 25	45 ± 37	< 0.01	
Reverse dipping pattern [*n* (%)]	5 (10)	17 (58)	< 0.01	
w-BPV > 11 mmHg [*n* (%)]	25 (49)	20 (68)	0.08	
SD-daytime SBP > 16 mmHg [*n* (%)]	4 (8)	10 (34)	0.02	
PPH [*n* (%)]	23 (46)	17 (58)	0.27	
Hypo-aw ^Δ15/24h^ [*n* (%)]	4 (8)	13 (44)	< 0.01	
Hypo-ep ^Δ15/24h^ (*n*.) (mean ± SD)	0.4 ± 0.6	3.4 ± 3.3	< 0.01	

*AF* autonomic failure, *LEDD* levodopa equivalent daily dose, *SBP* systolic blood pressure, *MBP* mean blood pressure, *DBP* diastolic blood pressure, *w*-*BPV* weighted blood pressure variability, *SD*-*daytime* *SBP* standard deviation of diurnal systolic blood pressure, *PPH* post-prandial hypotension, *Hypo*-*aw*^*Δ15/24h*^ awakening hypotension, *Hypo-ep*^*Δ15/24h*^ hypotensive episodes

**Fig. 1:**
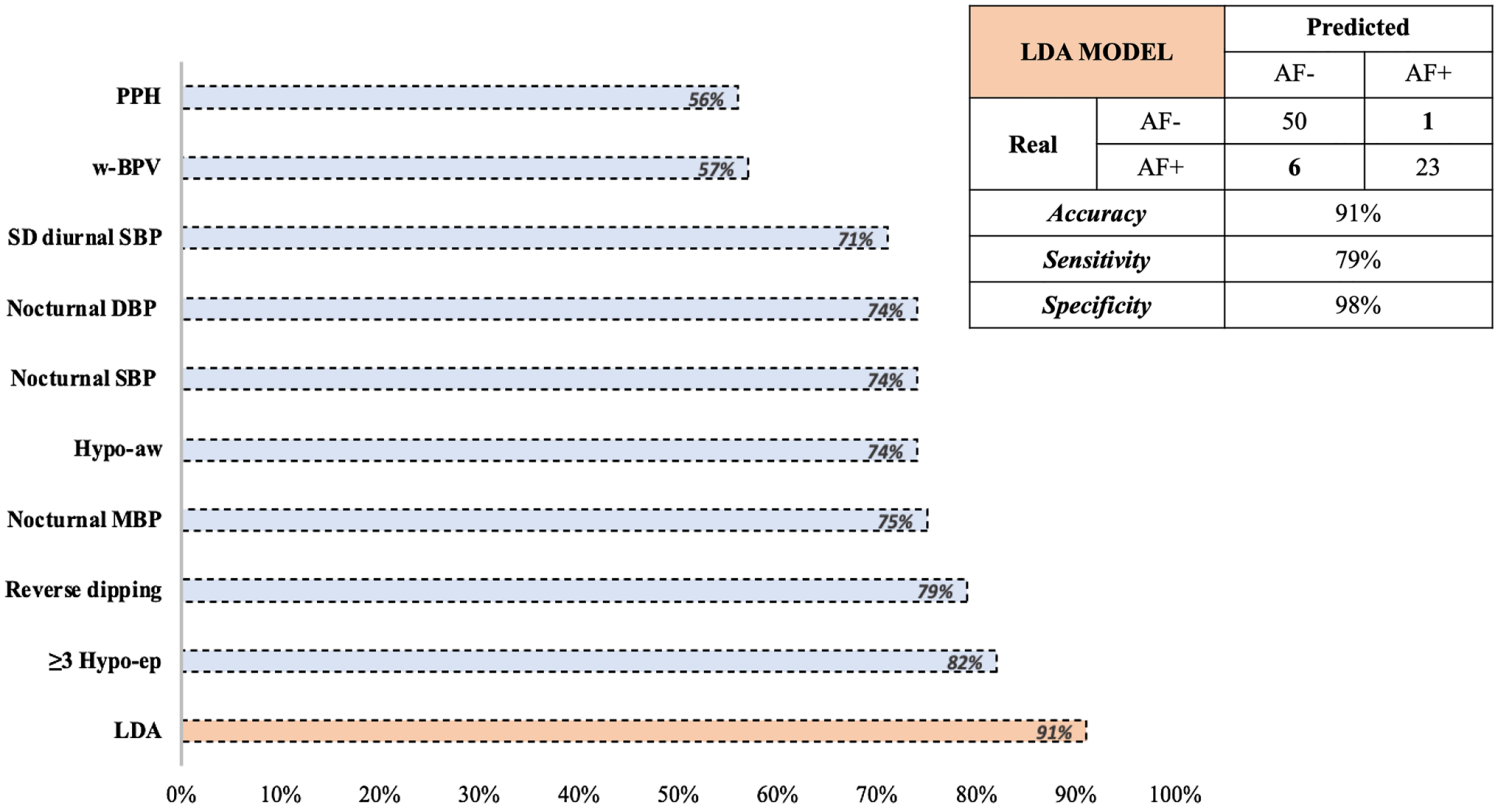
Accuracy of autonomic failure prediction. *AF* autonomic failure, *PPH* post-prandial hypotension, *w-BPV* weighted blood pressure variability, *SD* standard deviation, *SBP* systolic blood pressure, *DBP* diastolic blood pressure, *Hypo-aw*^*Δ15/24h*^ awakening hypotension, *MBP* mean blood pressure, *Hypo-ep*^*Δ15/24h*^ hypotensive episodes, *LDA* linear discriminant analysis

The LDA model was able to discriminate patients AF+ with 91.3% accuracy, 98.0% specificity, and 79.3% sensitivity, which was significantly higher than any of the ABPM variables considered individually (Table 2 and Fig. 1). The algorithm misdiagnosed only 6 patients with AF; among them, 1 with prevalent cardiovagal, 2 with prevalent adrenergic, and 3 with mixed AF.

**Table 2:** Prediction of autonomic failure through machine learning and single ABPM parameters

	Accuracy	AUC	Specificity	Sensitivity	PPV	NPV
*Machine learning (all ABPM variables)*						
LDA	91% (83–96)	/	98% (90–100)	79% (60–92)	96% (77–99)	89% (80–94)
*ABPM predictive variables (categorical)*						
≥ 3 Hypo-ep^Δ15/24h^	82% (71–90)	/	100% (93–100)	52% (33–71)	100%	77% (70–83)
Reverse dipping	79% (68–87)	/	90% (79–97)	59% (39–76)	77% (58–89)	79% (71–86)
Hypo-aw^Δ15/24h^	74% (63–84)	/	92% (80–98)	45% (26–64)	76% (54–90)	74% (67–80)
SD d-SBP (> 16 mmHg)	71% (60–81)	/	92% (81–98)	35% (18–54)	71% (46–88)	71% (65–77)
w-BPV (> 11 mmHg)	57% (46–68)	/	51% (37–65)	69% (49–84)	44% (36–54)	74% (61–84)
PPH	56% (44–67)	/	54% (39–68)	59% (39–76)	43% (33–53)	69% (58–79)
*ABPM predictive variables (continuous)*						
^a^Diurnal SBP	/	0.62 (0.49–0.75)	/	/	/	/
^a^Diurnal MBP	/	0.55 (0.42–0.69)	/	/	/	/
^a^Diurnal DBP	/	0.58 (0.46–0.71)	/	/	/	/
Nocturnal SBP (cut-off 123 mmHg)	74% (67–80)	0.72 (0.60–0.84)	90% (79–97)	45% (26–64)	72% (51–87)	74% (67–80)
Nocturnal MBP (cut-off 95 mmHg)	75% (64–84)	0.73 (0.61–0.85)	96% (87–99)	38% (21–58)	85% (57–96)	73% (67–78)
Nocturnal DBP (cut-off 75 mmHg)	74% (63–83)	0.67 (0.54–0.80)	92% (81–98)	41% (24–61)	75% (52–89)	73% (67–79)

Autonomic failure (AF+) was used as the outcome. The predictive power of each ABPM variable was calculated through a 2 × 2 contingency table for dichotomous variables (Hypo-aw^Δ15/24h^, ≥ 3 Hypo-ep^Δ15/24h^, postprandial hypotension, reverse dipping pattern, high weighted blood pressure variability) and through the ROC curve for continuous variables (diurnal and nocturnal blood pressure values). The accuracy of the continuous variables refers to the cut-point of the ROC curve with the best sensitivity–specificity compromise (123 mmHg for SBP, 95 mmHg for MBP, 75 mmHg for DBP)

LDA: linear discriminant analysis; ABPM: ambulatory blood pressure monitoring; Hypo-aw^Δ15/24h^: awakening hypotension; Hypo-ep^Δ15/24h^: hypotensive episodes; SD d-SBP: standard deviation of diurnal systolic blood pressure; w-BPV: weighted blood pressure variability; PPH: post-prandial hypotension; AUC: area under the curve; PPV: positive predictive value; NPV: negative predictive value; SBP: systolic blood pressure; MBP: mean blood pressure; DBP: diastolic blood pressure

^a^The ROC-curve output with diurnal BP value was obtained by inverting the outcome (AF−) to have an AUC greater than 0.5; accuracy metrics have not been reported being not significant

Further analyses were performed to determine the association of AF+ with individual variables while taking into consideration confounders, such as age, sex, disease duration and LEDD. Logistic regression analysis showed a strong association of AF+ with Hypo-aw^Δ15/24h^, ≥ 3 Hypo-ep^Δ15/24h^, and reverse dipping pattern (Table 3), while the association with an increased standard deviation of daytime systolic BP was not confirmed at the multivariate analysis. Nocturnal BP was also associated with AF+, with the mean BP value showing the strongest association (OR 1.09, *P* < 0.01) (Table 3B).

**Table 3:** Univariate and multivariate logistic regression analysis

A		Outcome	
ABPM predictive Variables (categorical)		Autonomic failure	
		Univariate analysis (IC 95%)	Multivariate analysis (IC 95%)
Hypo-aw^Δ15/24h^	OR	9.1 (2.6–32)	8.7 (2–37.4)
	*P* value	< 0.01	0.01
≥ *3* Hypo-ep^Δ15/24h^	OR	40.2 (5.8–78)	60.7 (12.1–108)
	*P* value	< 0.01	< 0.01
PPH	OR	1.6 (0.7–4.2)	1.4 (0.4–4.5)
	*P* value	0.28	0.57
Reverse dipping	OR	13 (4–42)	16.6 (3.2–87)
	*P* value	< 0.01	< 0.01
w-BPV (> 11 mmHg)	OR	2.3 (0.9–6)	1.4 (0.5–4.3)
	*P* value	0.09	0.57
DS daytime SBP (> 16 mmHg)	OR	6.1 (1.7–22.1)	3.8 (0.9–16)
	*P* value	< 0.01	0.06

B		Outcome	
ABPM predictive Variables (continuous)		Autonomic failure	
		Univariate analysis (IC 95%)	Multivariate analysis (IC 95%)
Diurnal SBP	OR	0.95 (0.9–1.01)	0.95 (0.89–1.01)
	*P* value	0.06	0.06
Diurnal MBP	OR	0.96 (0.9–1.01)	0.96 (0.89–1.03)
	*P* value	0.14	0.24
Diurnal DBP	OR	0.97 (0.91–1.02)	0.97 (0.91–1.04)
	*P* value	0.24	0.41
Nocturnal SBP	OR	1.07 (1.03–1.11)	1.06 (1.01–1.12)
	*P* value	< 0.01	0.01
Nocturnal MBP	OR	1.09 (1.04–1.15)	1.08 (1.02–1.15)
	*P* value	< 0.01	0.01
Nocturnal DBP	OR	1.08 (1.03–1.14)	1.07 (1.01–1.13)
	*P* value	< 0.01	0.03

Autonomic failure (AF+) was used as dependent variable (outcome). In univariate analysis, the independent variables were Hypo-aw^Δ15/24h^ (awakening hypotension), ≥ 3 Hypo-ep^Δ15/24h^ (hypotensive episodes), reverse dipping, w-BPV (weighted blood pressure variability), DS-daytime SBP (standard deviation of daytime systolic blood pressure), diurnal and nocturnal SBP (systolic blood pressure), diurnal and nocturnal MBP (mean blood pressure), diurnal and nocturnal DBP (diastolic blood pressure). In multivariate analysis age, sex, disease duration and LEDD (levodopa equivalent daily dose) were used as potential confounding variables

## Discussion

In this study, the diagnostic performance of a supervised learning algorithm employing ABPM recordings to diagnose AF in patients with PD was assessed. The model was able to discriminate AF with 91.3% accuracy, much higher than any of the other ABPM variables considered independently. In particular, while individual ABPM parameters, such as ≥ 3 hypotensive episodes, awakening hypotension, reverse dipping, or increased nocturnal BP could identify AF with relatively good specificity, they were all limited by low sensitivity (< 60%), hampering their potential as a screening tool.

Clinical manifestations of AF encompass both short- and long-term dysregulations in BP regulatory mechanisms. The former include OH and SH, the latter include nocturnal hypertension, abnormal circadian rhythm, and increased BPV [1]. SH and reverse dipping, in particular, have been associated with hypertensive end-organ damage and worse clinical prognosis in patients with PD [4, 24, 25]. Still, the extent to which a correction of these hemodynamic abnormalities might result in clinical benefit remains to be clarified. The introduction of a machine-learning-based algorithm of 24-h ABPM bears the promise to help understand the complex interaction between hemodynamic parameters and functional outcomes. A deeper understanding of BP dysregulation in AF will allow detecting profiles of BP abnormalities with a higher risk of adverse outcomes and inform the selection of treatment priorities (e.g., balancing risk and benefits of better control of SH at the expense of higher burden of OH versus allowing higher supine and nocturnal BP to mitigate OH) [26, 27].

The present analyses confirm the previous finding that hypotensive episodes and reverse dipping are accurate markers of AF in PD [9, 20], while increased BPV seems to be less effective in predicting AF, despite the multiple hypotensive episodes (expected to increase BPV) observed in this patient population. While this result partly conflicts with a previous study [10] suggesting that exaggerated SD of diurnal systolic BP could be used to detect primary or secondary AF, the authors did not confirm the association between AF and increased SD-SBP when the PD status and dopaminergic treatment were included in the multivariate analysis. This suggests that AF in PD (and possibly other forms of primary AF) may be characterized by a peculiar BP profile, different from the one observed in secondary AF.

The strength of this study is the innovative approach involving machine learning for the detection of AF, that demonstrated high accuracy and specificity, and relatively high sensitivity.

The assessment of patients in their real-life environment allows exploring the everyday BP profiles, which may be more informative on the risk of organ damage development than the standardized but artificial values obtained through CART. Several limitations, however, should also be considered in the interpretation of the results.

First, the number of patients with AF was relatively low due to the stringent exclusion criteria, aiming at limiting confounders related to additional pharmacological treatment or concurrent clinical conditions; to reduce this bias, patients were carefully selected without vasoactive medications or known cardiovascular comorbidities, or severe cognitive impairment. Second, dopaminergic drugs have not been withheld during CART and ABPM, to assess BP fluctuations in a real-life environment. Still, the impact of dopaminergic drugs may have influenced the BP recordings. To that extent, the finding that LEDD values were not significantly different among groups and most associations remained significant after adequate correction in multivariate analysis seems reassuring. Third, the possibility exists that ABPM could better capture adrenergic impairment, thus limiting the diagnosis of AF with a prominent cardiovagal impairment, although the analysis of the 6 misidentified patients does not seem to confirm this hypothesis. Fourth, the variability in each individual patient’s day schedule might have influenced the ABPM recordings, as those with greater motor disability are less likely to engage in strenuous physical activities or prolonged standing.

This should be considered as a pilot study, but a wide range of future applications for machine learning in the field of ABPM can be easily envisioned. The machine learning approach needs to be tested and validated on larger samples, evaluating the possibility to discriminate patients with prevalent cardiovagal vs. adrenergic vs. mixed autonomic impairment, with associated clinical implications. It seems reasonable to assume that patients with prevalent cardiovagal impairment should display a peculiar BP profile, since adrenergic vasoconstriction is usually preserved while HR variations are minimal or absent. Similarly, one would expect that patients with prevalent adrenergic impairment, with minimal vasoconstrictive function but preserved compensatory shifts in HR, could be differentiated by those with mixed AF. The extent to which machine learning applied to ambulatory recordings of blood pressure and heart rate can assist in detecting distinctive patterns of blood pressure dysregulation with potentially relevant clinical implications remains to be clarified. In the meantime, these data suggest that this technology can be successfully applied to ABPM recordings to diagnose AF when CART is not easily available or difficult to obtain, favoring more appropriate referrals to a second-level CART evaluation, with the main advantage of lowering healthcare costs, improving the appropriateness of referrals, and providing an additional, real-life, measure of circadian blood pressure fluctuations. Additional possible applications include monitoring the efficacy of treatments aiming at correcting OH without resulting in excessive SH.
